# Effects of Xinjiaxiangruyin on the TLR7 pathway in influenza virus-infected lungs of mice housed in a hygrothermal environment

**DOI:** 10.1186/s13020-019-0256-7

**Published:** 2019-09-27

**Authors:** Ying-Jie Fu, Yu-Qi Yan, Xiao Zheng, Shan-Shan Shi, Sha Wu, Zhen-You Jiang

**Affiliations:** 1 Department of Microbiology and Immunology, School of Basic Medical Sciences, Jinan University, Guangzhou, 510632 Guangdong China; 2 Institute of Medical Microbiology, Jinan University, Guangzhou, 510632 Guangdong China; 3 Guangdong Province Engineering Research Center for Antibody Drug and Immunoassay, Guangzhou, 510632 Guangdong China

**Keywords:** Traditional Chinese Medicine, Hygrothermal environment, Influenza A virus, TLR7 pathway, Xinjiaxiangruyin

## Abstract

**Background:**

To investigate the effects and immunological mechanisms of the traditional Chinese medicine Xinjiaxiangruyin on controlling influenza virus (FM1 strain) infection in mice housed in a hygrothermal environment.

**Methods:**

Mice were housed in normal and hygrothermal environments, and intranasally infected with influenza virus (FM1). A high-performance liquid chromatography fingerprint of Xinjiaxiangruyin was used to provide an analytical method for quality control. Real-time quantitative polymerase chain reaction (RT-qPCR) was used to measure messenger RNA expression of Toll-like receptor 7 (TLR7), myeloid differentiation primary response 88 (MyD88), and nuclear factor-kappa B (NF-κB) p65 in the TLR7 signaling pathway and virus replication in the lungs. Western blotting was used to measure the expression levels of TLR7, MyD88, and NF-κB p65 proteins. Flow cytometry was used to detect the proportion of Th17/T-regulatory cells.

**Results:**

Xinjiaxiangruyin effectively alleviated lung inflammation in C57BL/6 mice in hot and humid environments. Guizhimahuanggebantang significantly reduced lung inflammation in C57BL/6 mice. The expression of TLR7, MyD88, and NF-κB p65 mRNA in lung tissue of WT mice in the normal environment, GZMHGBT group was significantly lower than that in the model group (P < 0.05). In WT mice exposed to the hot and humid environment, the expression levels of TLR7, MyD88, and NF-κB p65 mRNA in the XJXRY group were significantly different from those in the virus group. The expression levels of TLR7, MyD88, and NF-κB p65 protein in lung tissue of WT mice exposed to the normal environment, GZMHGBT group was significantly lower than those in the model group. In WT mice exposed to hot and humid environments, the expression levels of TLR7, MyD88, and NF-κB p65 protein in XJXRY group were significantly different from those in the virus group.

**Conclusion:**

Guizhimahuanggebantang demonstrated a satisfactory therapeutic effect on mice infected with the influenza A virus (FM1 strain) in a normal environment, and Xinjiaxiangruyin demonstrated a clear therapeutic effect in damp and hot environments and may play a protective role against influenza through downregulation of the TLR7 signal pathway.

## Background

Influenza virus infection, whether seasonal or pandemic, can cause severe health problems, often leading to pneumonia [1]. The death toll of influenza epidemics is between 250,000 and 500,000 [2]. The latest influenza pandemic was caused by a swine-origin H1N1 virus, which is a triple reassortment of human, avian, and swine strains [3]. The frequent reassortment of influenza virus may cause high mortality and overburden the healthcare system [4]. Innate immunity is the body’s first line of defense against pathogens, and toll-like receptors (TLRs) are important pattern recognition receptors (PRRs). PRR recognition of microbial pathogens is required to initiate the natural immune response. PRRs recognize a variety of conserved molecules of the pathogen, known as the pathogen-associated molecular pattern (PAMP) [5]. TLR7, which is highly expressed in human plasmacytoid dendritic cells [6], is a member of the TLR family that recognizes single-stranded RNA viruses [7] such as the influenza virus. After recognition of PAMP, TLR7 recruits specific linker molecules that bind to TIR domains, such as myeloid differentiation factor 88 (MyD88), and contain TIR structures, and can induce the interferon-β linker molecule (TRIF) and then, through a series of signal conduction, eventually lead to cell production of inflammatory factors, type I interferons, chemokines, and antibacterial peptides [8]. Finally, it induces nuclear transfer of NF-κB and activates a signaling pathway that produces an inflammatory response.

For thousands of years, Chinese medicine has accumulated a wealth of valuable experience in combating the plague. In the fight against severe acute respiratory syndrome, Chinese medicine practitioners actively participated in clinical treatment and research at various levels, and in various aspects such as prevention, treatment, and late recovery. The effectiveness and safety of an integrated traditional Chinese and western medicine method has been fully recognized by the World Health Organization. The successful prevention and treatment of influenza using Chinese medicine has also been recognized worldwide.

Yinqiaosan (YQS) is a representative medicine of the cool acrid exterior-resolving method. This medicine has the effect of pungent cooling and detoxification. It is used to treat infectious diseases such as influenza, acute tonsillitis, angina, herpes, mumps, and other viral infections [9]. Xinjiaxiangruyin (XJXRY) was created by Wu Jutong, a warm disease scientist of the Qing Dynasty. Its composition is exquisite, and the types of drugs used are small and light in dosage. However, as a representative medicine for clearing dampness and relieving superficies, it is often used in the clinical treatment of summer influenza and to good effect. Guizhimahuanggebantang (GZMHGBT) is a representative medicine of the warm acrid exterior-resolving method. GZMHGBT has pharmacological effects and properties including antipyretic, analgesic, anti-flu, anti-inflammatory, anti-asthmatic, immune intervention, and anti-allergy [10]. Clinically, it is mainly used for the treatment of colds, upper respiratory tract infections, allergies, and skin musculature.

The present study explored the effects of YQS, GZMHGBT and XJXRY on the TLR7 pathway and immune balance of T-helper (Th) 17 and T-regulatory (Treg) cells in mice infected with H1N1 (FM1 strain) in hot and humid, and normal environments. The possible mechanism of action of Chinese medicine against influenza in these environments was investigated.

## Materials and methods

### Information of experimental design and resources

The information regarding the experimental design, statistics, and resources used in this study are attached in the minimum standards of reporting checklist.

### Instruments

High-performance liquid chromatograph (1260, Agilent Technologies Inc); electronic balance (AL104, Mettler Toledo Instruments, Co., Ltd., Shanghai, China);

Desktop High Power CNC Ultrasonic Cleaner (KQ-800KDE, Keqiao Ultrasonic Equipment, Co., Ltd., Dongguan, China); Vortex Mixer (QL-901, Qilin Medical Instrument Factory, Haimen, Jiangsu, China); Small Vertical Electrophoresis Cell (Mini-Protean Tetra 1658001), Basic Electrophoresis System (PowerPac Basic164-5050) and Small-scale transfer system core (Mini Trans-Blot 1703935) were purchased from Bio-Rad, USA; optical microscope (BX51, Olympus Optical, Co., Ltd., Japan); PCR Thermal Cycler (846X070241, Analytik Jena AG, Germany); Real-Time PCR System (CFX96™, Bio-Rad, USA); flow cytometer (BD FACSVerse™, Bio-Rad, USA).

### Medicines

Yinqiaosan (i.e., YQS) (15 g Fructus Forsythiae, 15 g Flos Lonicerae, 9 g Radix Platycodonis, 9 g Herba Menthae, 6 g Herba Lophather, 5 g Radix Glycyrrhizae, 6 g Herba Schizonepetae, 6 g Fermented soybean, 6 g Fructus arctii, 10 g Rhizoma Phragmitis); Xinjiaxiangruyin (i.e., XJXRY) (6 g Herba Moslae, 9 g Flos Lonicerae, 9 g Dolichos, 6 g Cortex Magnoliae Officinalis, 6 g Fructus Forsythiae). Guizhimahuanggebantang (i.e., GZMHGBT) contained equal part of Mahuang Tang (9 g Herba Ephedrae, 6 g Ramulus Cinnamomi, 9 g Semen Armeniacae Amarum, 6 g Radix glycyrrhizae preparata) and Guizhi Tang (9 g Ramulus Cinnamomi, 9 g Radix Paeoniae Alba, 6 g Radix Glycyrrhizae, 9 g Rhizoma Zingiberis Recens, 3 g Jujube), which were dissolved in water and combined. These herbs were all Chinese patent medicine granules, which were purchased from China Resources Sanjiu Medical & Pharmaceutical Co., Ltd. Oseltamivir Phosphate Capsules (i.e., Oseltamivir) was obtained from Yichang Yangtze River East Sunshine Pharmaceutical Ltd. (Lot H20065415).

### Reagents

Chromatographically pure acetonitrile and methanol, ultrapure water, and other analytically pure reagents. Chlorogenic acid (Lot 171110; purity 99.37%); Forsythin (Lot 171103; purity 99.01%); Thymol (Lot 171210; purity 98.59%); Magnolol (Lot 171126; purity 99.29%), which were purchased from Shengshi Kangpu Chemical Technology Research Institute (Beijing, China). PrimeScript RT reagent kits (Lot: RR047A, TaKaRa, Japan), SYBR Premix EX Taq II (Lot: RR820A, TaKaRa, Japan). DEPC Water, (Sigma, USA); TLR7 Rabbit mAb (Lot: 2633S), MyD88 Rabbit mAb (Lot:4283S), NF-kappa B p65 Rabbit mAb (Lot: 8242S), GAPDH Rabbit mAb (Lot: 2118S) were purchased from CST, USA; The mouse lymphocyte separation solution, Goat anti-rabbit secondary antibody, BCA Protein Quantitative Kit, and potent ECL kits were all products of Multi Sciences Co., Ltd. in Hangzhou, China. CD4-PE (Phycoerythrin) (Lot: 4343463); CD25-APC (Allophycocyanin) (Lot: E07106-1634); IL-17-FITC (Lot: 4291420); Foxp3-PE-cyanine 5.5 (Lot: E16149-104), eBioscience™ Foxp3/Transcription Factor Staining Buffer Set Kit (Lot: 4343791) were purchased from eBioscience Inc., USA.

### Viruses

Virus strain: Influenza virus A (FM1/1/47 strain, mouse adapted), type A influenza virus FM1 murine lung adapted strain (FM1), − 80 °C cryopreserved, provided by the Department of Microbiology and Immunology, School of Basic Medical Sciences, Jinan University. After two passages of resuscitation, the hemagglutination titer was 1:640. Mortality was assessed at 14 days after statistical viral infection at different concentrations using a multiple dilution method; the virus concentration at 20% of the mouse hemagglutination titer (1:40), with 50 μL used to infect the mice.

### Animals and groups

Animal experiments were performed with the approval and supervision of the Experimental Animal Ethics Committee of Jinan University (Guangdong, China). SPF-grade normal C57BL/6 wild-type mice were purchased from the Experimental Animal Center of Guangdong Province (production license SCXK [Guangdong] 2013-0002). The TLR7^−/−^ mice were purchased from The Jackson Laboratory, USA. All knockout mice were backcrossed to the germline of C57BL/6 mice over 10 generations. Reproduction and breeding of TLR7^−/−^ mice were performed in the SPF environment at the Animal Experimental Center of Jinan University. Six to eight-week-old female experimental animals were placed in artificial climate chambers: normal environment (temperature, 20 ± 1 °C; humidity, 50 ± 5%); and hot and humid environment (temperature, 33 ± 1 °C; humidity, 90 ± 5%). The illuminance was 3000 Lx and 0 Lx each 12 h cycle. Mice were reared on a cluster basis. Thirty-Six C57BL/6 mice (33 °C, wild type [WT]) and TLR7^−/−^ mice (33 °C, TLR^−/−^) were housed in a hot and humid environment and randomized to six animals per group. After infection with the virus, the mice were placed in the artificial climate chambers for 12 h each day and housed in normal climate chambers for the rest of the day until the end of the experiment. The animals had ad libitum access to food and water. There were six mice in each of the following groups: blank (control); model (virus); virus + oseltamivir; virus + XJXRY; virus + GZMHGBT; and virus + YQS. Thirty-six (20 °C, WT) C57BL/6 mice of the same age were housed in a normal environment similar to the control group.

### Establishing a flu mouse model for the humid and hot environment

On day 11 of modeling in a hot and humid environment, both TLR7^−/−^ and WT C57BL/6J mice were infected with the influenza virus (FM1). The mice were first anesthetized with 6% chloral hydrate solution, and were infected intranasally with 50 μL of the FM1 strain of the influenza virus. The normal control group was given an equal volume of sterile 0.9% NaCl solution by intranasal instillation. None of the nasally infected mice died. General living conditions and body weight of the mice were observed and recorded daily.

### Anti-influenza virus experiment

On day 11 day of modeling, the mice were infected with FM1 virus. Twenty-four hours after the mice were infected, each group of animals was treated with the corresponding drug, with dose calculated according to body weight. The dose used in mice is equivalent to 9.1 times that of humans. The treatment group was given 0.4 mL/day, with continuous intragastric administration for 5 days (12–16 days): YQS, 560 mg/mL; GZMHGBT, 350 mg/mL; XJXRY, 230 mg/mL; and oseltamivir 1.5 mg/mL. An equal amount of double distilled water was given to the normal group and the virus group. The body weight of all mice was recorded on day 17 after infection, and lung tissues and spleen were immediately obtained after the mice were euthanized.

### HPLC fingerprint and qualitative analysis of XJXRY

Chromatographic conditions. Columns: Cosmosil 5C18-MS-II (4.6 ID × 250 mm) Manf. No. K63663. Mobile phase: Acetonitrile (A)–0.1% H_3_PO_4_ (B); Flow rate: 1.0 mL/min; column temperature: 30 °C; detection wavelength: 280 nm; injection volume: 10 μL, the gradient elution program is shown in Table 1 [11].

**Table 1:** The table of gradient elution

Time (min)	0	5	15	30	42	66	75	85	110	111	116	120	130
A%	5	5	10	15	17	20	30	50	70	95	95	5	5
B%	95	95	90	85	83	80	70	50	30	5	5	95	95

### Preparation of the reference solution

3 mg of chlorogenic acid, forsythia, thymol, and magnolol were transferred to a brown volumetric flask and methanol was added to prepare a 0.5 mg/mL standard solution.

### Preparation of the test product solution

**Fig. 1:**
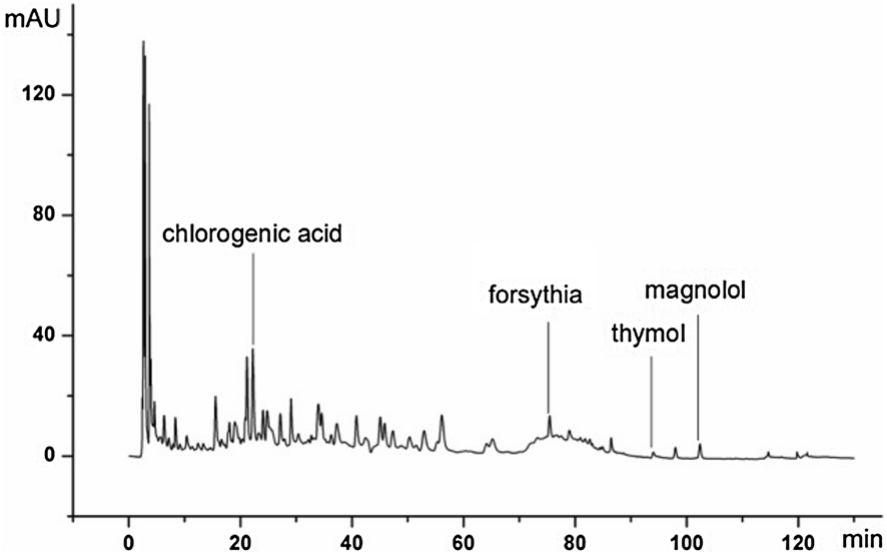
The fingerprints of Xinjiaxiangruyin; peak identity, chlorogenic acid; forsythia; thymol; magnolol

According to the ancient decocting method, lentil flower soup was obtained, cold to room temperature, and a precise quantity (12.5 mL) was transferred to a 50 mL tapered bottle. Fine particles of honeysuckle, forsythia, Magnolia, and Elsholtzia were mixed in a conical bottle and mixed with the lentil flower soup. Add the 12.5 mL methanol to the cone bottle again, weighing and ultrasonic cleaning 30 min (power 100 W, 30 °C). When it is cooled to room temperature, and then weigh it; 50% methanol was used to fill the lost weight, shake well, filter, and remove the filtrate through 0.45 µm porous filter membrane to obtain the test product solution (Fig. 1).

### Changes in body weight, survival rate, and lung index

The weight of each mouse was measured and recorded at the same time point every day. Mice were subjected to environmental modeling from day 1 to day 11 and weight was recorded. Calculation formula: The change on weight = day n weight/Original weight × 100%. The mice were infected with influenza virus from day 11. The weight of mice from day 12 to day 17 was recorded. Calculation formula: The change on weight = day n weight/day 11 weight × 100%. The changes of symptoms, water and food intake, hair color, activity, death condition and so on were observed twice a day.

The mice were euthanized on day 6 when they were exposed. The lung organs were removed and the adipose tissue on the lung tissue was removed. The lung tissue was washed with sterile PBS, blotted dry with filter paper, and total lung weight was measured. Lung index was calculated based on the body weight of each mouse. Calculation formula: lung index = day 17 lung weight/day 17 body weight × 100%.

### Preparation of mouse tissue specimens

Six days after influenza virus infection, the specimens were observed and preserved. Rapid isolation of lung tissue, fresh left lung, freezing at − 80 °C, RT-qPCR, and western blot detection. The middle lobe of the right lung was fixed and paraffin-embedded for sectioning. Hematoxylin and eosin staining was performed. At the same time, separation of splenic cells was performed using flow cytometry.

### Hematoxylin and eosin staining

On day 6 after infection, the mice were dissected to remove all lung tissue. The right middle lobe was isolated and fixed in 4% paraformaldehyde solution, embedded in paraffin, and cut into 5-μm thick sections. Two paraffin sections were randomly selected from each mouse and hematoxylin–eosin staining was performed. The morphological changes in the lung tissues of mice in each group were observed under a light microscope and photographed.

### RT-qPCR

Total RNA from the left lung tissue was extracted using Trizol and single-stranded complementary DNA was synthesized using a commercially available reverse transcription kit. All primers are shown in Table 2, which were designed and synthesized by Shanghai Generay Biotech Co., Ltd.

**Table 2:** Primers used for RT-qPCR studies

Gene	Forward (5′ to 3′)	Reverse (5′ to 3′)
FM1	GACCAATCCTGTCACCTCTGAC	AGGGCATTNTGGACAAAGCGTCTA
TLR7	GGGTCCAAAGCCAATGTG	TGTTAGATTCTCCTTCGTGATG
MyD88	CGATTATCTACAGAGCAAGGAATG	ATAGTGATGAACCGCAGGATAC
NF-κB	ATTCTGACCTTGCCTATCTAC	TCCAGTCTCCGAGTGAAG
GAPDH	CTGAGCAAGAGAGGCCCTATCC	CTCCCTAGGCCCCTCCTGTT

The PCR reaction (20 μL): PCR forward primer (10 μM) 0.8 μL, PCR reverse primer (10 μM) 0.8 μL, SyBr Premix Ex Taq II(2*1) 10 μL, complementary DNA template 2 μL, dd H_2_O 6.4 μL. PCR reaction conditions: 95 °C for 30 s; 95 °C for 5 s, 60 °C for 30 s, 40 cycles; 95 °C for 10 s. Melting curve analysis: temperature 65–95 °C. Repeat 3 times for each sample. A real-time quantitative PCR instrument (CFX96, Bio-Rad, Temecula, CA, USA) was used for fluorescence quantification experiments. The Ct value of each determination was recorded and GAPDH was used as the internal reference. The relative mRNA expression of the sample was calculated according to the 2^−∆∆Ct^ method. ∆Ct = Ct_target gene_ − Ct_reference gene_, ∆∆Ct = ∆Ct_experimental group_ − Ct_control group_. Each sample was measured three times and averaged, and qPCR results represented three independent experiments.

### Expression of TLR7, MyD88, and NF-kB p65 protein detected by western blot

Homogenates (40 mg) were collected from lung tissue stored at − 80 °C, thawed for 15 min at 4 °C and centrifuged at 12,000 rpm, and the supernatant was collected. Bovine serum albumin (BSA) was used as standard, and a commercially available BCA protein quantitative kit was used to determine protein content. An equal amount (35 μg) of the protein contained in each sample was decomposed in a 10% Tris–glycine SDS polyacrylamide gel. After electrophoresis, the separated protein bands were wet transferred to polyvinylidene fluoride (PVDF) membranes. Nonspecific binding membranes were blocked with 5% skim milk at room temperature for 1 h. The PVDF membrane was incubated with primary antibody (1:1000) and incubated overnight at 4 °C. Diluted secondary antibody was added at a ratio of 1:5000 and incubated for 1 h at room temperature. The immunodetection protein was visualized using an electroluminescence kit and the Alliance gel imaging system, and analyzed using image J software. The ratio of the target protein optical density value and the internal reference protein GAPDH optical density value was considered to represent the relative expression of the target protein.

### Flow cytometry

The ratio of subcellular Th17/Treg (CD4+IL-17+/CD4+CD25+Foxp3+) of T lymphocytes in mice was determined using flow cytometry. Peripheral blood mononuclear cells were isolated from mouse spleens using a lymphocyte separation solution according to manufacturer’s instructions. The regulatory cell concentration was approximately 1 × 10^6^ cells/mL. For flow cytometry, the cells were labeled with a monoclonal antibody conjugated to fluorescein (CD4 mAb, IL-17 mAb, CD25 mAb, FOXP3 mAb) on a FACSVerse flow cytometer (Becton–Dickinson Bioscience NCS, Franklin Lakes, NJ, USA) and were tested and analyzed using FlowJo version 10 (FlowJo, Ashland, OR, USA).

### Statistical methods

Experimental data were processed and analyzed using SPSS version 13.0 (SPSS Inc, Chicago, IL, USA). All indicators were expressed as mean ± standard deviation. Multiple comparisons were carried out by one-way ANOVA followed by Bonferroni’s post hoc test or one-way ANOVA with repeated measures and Bonferroni test for multiple comparisons. All tests were two-tailed and P values less than 0.05 were considered statistically significant. Differences with P < 0.05 were considered to be statistically significant.

## Results

### Comparison of general state, weight change, and lung index of mice

Two days after influenza virus infection (day 13), the clinical symptoms of mice in each treatment group and virus model group became apparent, with varying degrees of weight loss, cough, fur loss, and contractures. On day 15, mice had typical flu symptoms, including hair discoloring, towering hair, curled up, arch, loss of appetite, faint. The control group exhibited a good mental state, agile movement, and natural weight gain. In the normal environment, mice in the oseltamivir, GZMHGBT were in good condition. However, in the hot and humid environment, the mice in the XJXRY and oseltamivir groups were in a better state than the virus group.

**Fig. 2:**
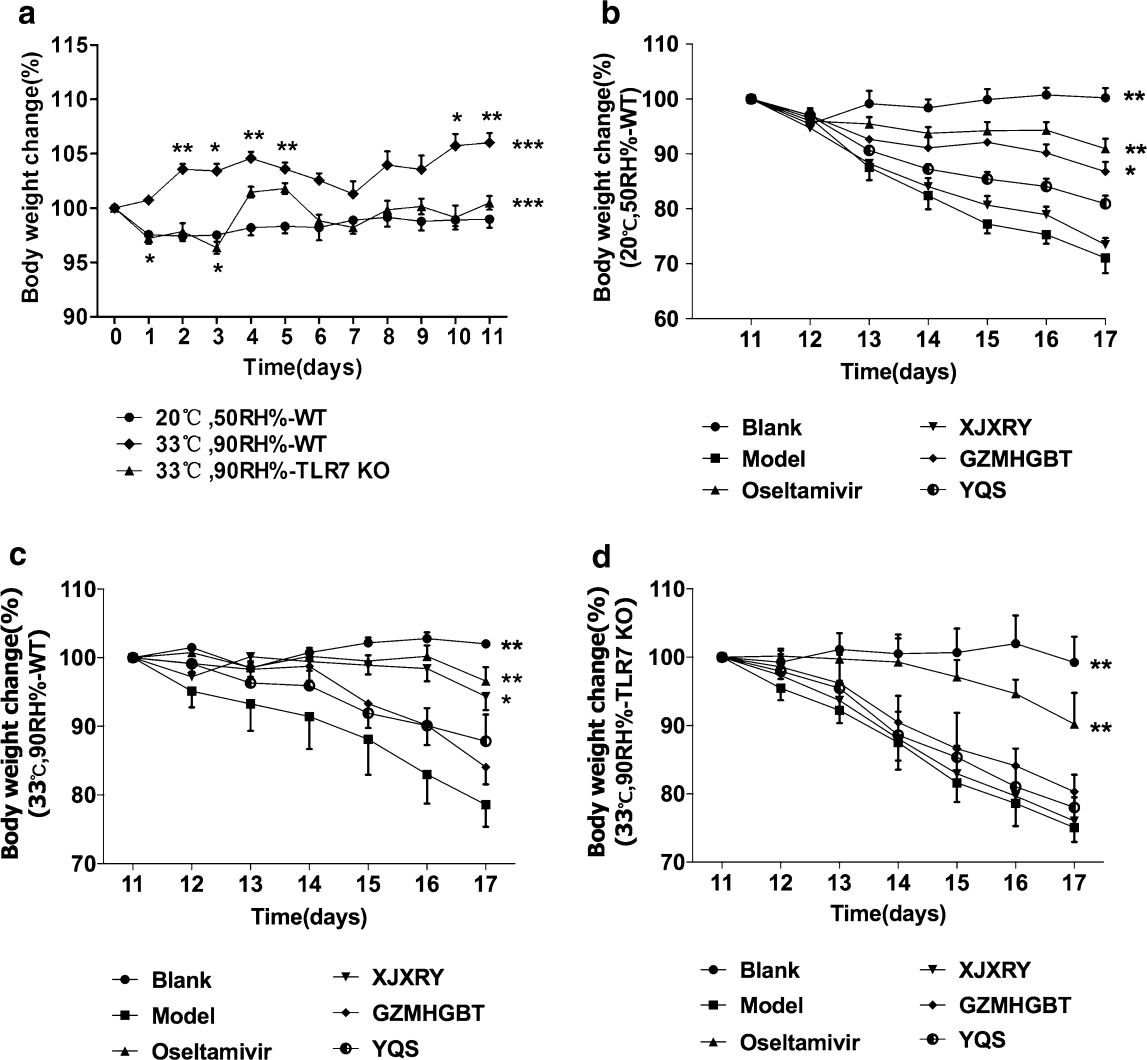
Effect of environment on body weight of mice and effects of three TCM compounds on body weight loss and lung index of mice infected with influenza virus. **a** Changes of body weight in mice for 11 days of environmental modeling. 20 °C, 50RH%-C57BL/6 wild type (**b**), 33 °C, 90RH%-C57BL/6 wild type (**c**) and 33 °C, 90RH%- TLR7^−/−^ (**d**) mice were infected with 50 μL FM1 virus on day 11 and then were gavaged with distilled water, containing oseltamivir, XJXRY, GZMHGBT or YQS daily 24 h after virus infection, respectively. Changes in body weight of each mouse were recorded. Statistical analysis was performed by one-way ANOVA with repeated measures (**a**) and one-way ANOVA (**b**–**d**). P* = P value for Bonferroni correction. *P < 0.05, **P < 0.01, ***P < 0.001

The mice were modeled in normal environment and hot and humid environment for 11 days (Fig. 2a). We can find that the weight of wild type mice in normal environment has almost no change (F_(11,385)_ = 1.892, P = 0.061). However, the weight of wild type (F_(11,385)_ = 10.624, P < 0.0001) and TLR7^−/−^ mice (F_(11,385)_ = 12.108, P < 0.0001) had obvious fluctuation in hot and humid environment. TLR7^−/−^ mice lost weight, but returned to the original weight on the 11th day.

However, except for the control group, the body weights of the other groups exhibited significant changes by day 17. Under normal circumstances (F_(5,28)_ = 34.78, P < 0.0001), the body weight of the mice on day 17 of the GZMHGBT, YQS, and oseltamivir groups decreased to 86.78%, 80.93%, 90.96% of the body weight on day 11, respectively (Fig. 2b). In the hot and humid environment (F_(5,26)_ = 12.35, P < 0.0001), the body weight of mice on day 17 in the XJXRY, and oseltamivir decreased to 92.34%, 96.61% of the body weight on day 11, respectively (Fig. 2c). In TLR7^−/−^ mice (F_(5,25)_ = 27.35, P < 0.0001), except for the control group, there was no difference between the other groups and the virus group (Fig. 2d).

**Fig. 3:**
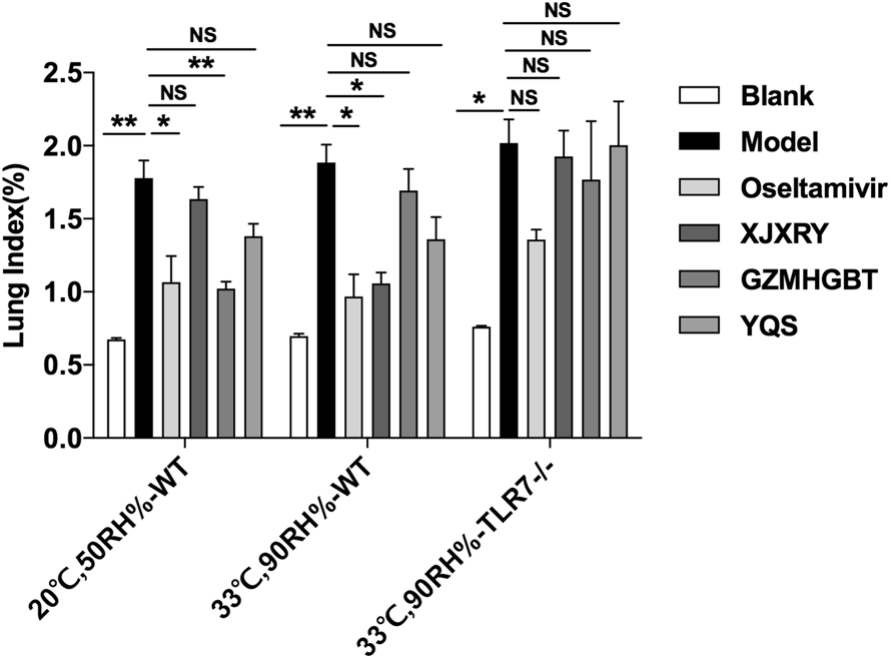
Effects of three TCM compounds on lung index of mice infected with influenza virus. The body weight of mice was monitored every day for 17 days. The mice were sacrifice on day 17, and the total lung were completely removed and weighed. Lung index = day 17 lung weight/day 17 body weight × 100%. Statistical analysis was performed by one-way ANOVA. P* = P value for Bonferroni correction. *P < 0.05, **P < 0.01, ***P < 0.001.

Compared with the control group, the lung index of the other groups increased to varying degrees. As a positive control drug, oseltamivir was not associated with a significant increase in lung index, which was different from that of the model group (P < 0.05). Statistical analysis revealed that in the hot and humid environment (F_(5,26)_ = 13.99, P = 0.0001), the WT mice XJXRY and the model group were different (P < 0.05). In the TLR7^−/−^ mice (F_(5,25)_ = 6.25, P = 0.002), there was no statistical difference between the XJXRY and the virus group (Fig. 3).

### Viral load of lung tissue

**Fig. 4:**
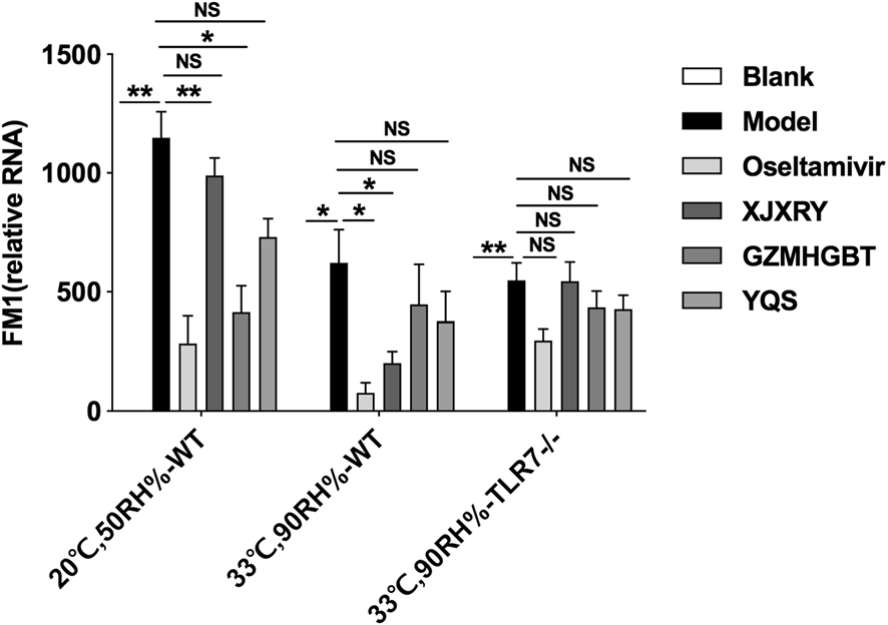
The FM1 viral load of the lung tissue of mice infected with influenza virus for 6th days. Statistical analysis was performed by one-way ANOVA. P* = P value for Bonferroni correction. *P < 0.05, **P < 0.01, ***P < 0.001

Viral load in mouse lung tissue was assessed using RT-qPCR. The expression of FM1 RNA in the mouse lung tissue of the virus model group was significantly higher than that of the control group (P < 0.05). Under normal circumstances (F_(5,28)_ = 23.45, P < 0.0001), compared with the model group, the dose of oseltamivir group, GZMHGBT decreased significantly (P < 0.05). In the hot and humid environment (F_(5,26)_ = 8.91, P < 0.0001), there was a significant difference between the oseltamivir and XJXRY groups with the virus group (P < 0.05). In TLR7^−/−^ mice (F_(5,25)_ = 11.37, P = 0.0003), the viral load in the model group was significantly higher than that in the control group (P < 0.001) (Fig. 4).

### Pathological changes in lung tissue of mice in each group

On day 17, there were no abnormal changes in the structure of lung tissue in the control group, and the alveolar structure was clearly visible. In contrast, the alveoli in the model group exhibited a large number of inflammatory cell infiltrates, the most inflammatory lesions, severe alveolar septal thickening, interstitial edema, and massive mononuclear cell infiltration. In the WT mice, the lesions in the lungs in the normal environment of the GZMHGBT and in the humid environment of the XJXRY groups were lighter than those in the virus group, the alveolar walls were thinner, and there were only a few monocytes in the alveoli and lymphocytes.

**Fig. 5:**
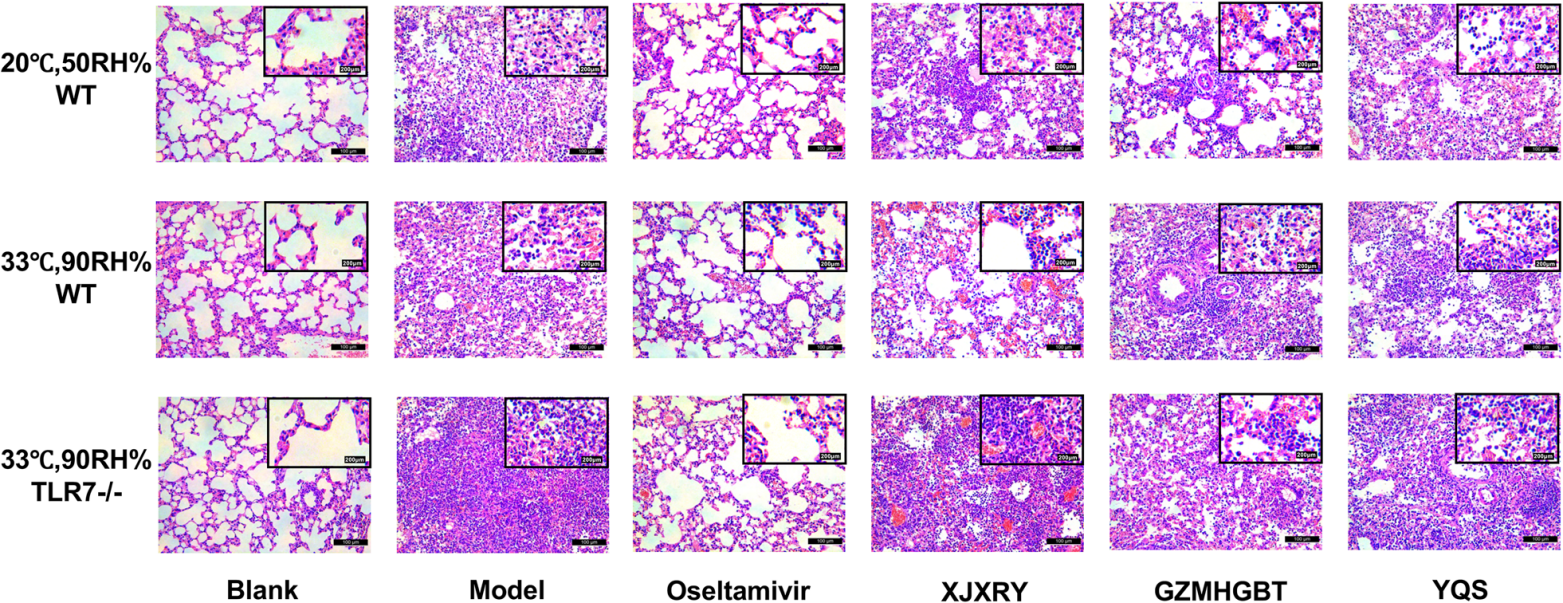
Lung histopathology in 20 °C, 50RH%-wild type, 33 °C, 90RH%-wild type and 33 °C, 90RH%-TLR7^−/−^ mice infected with influenza FM1. The lungs were collected on day 17, and sections were prepared for histopathological analysis. This figure shows representative results of experiments with three mice in each group. Bars = 100 μm

In the TLR7^−/−^ mice, the destruction of the alveolar structure was increased in the XJXRY group, and a large volume of edema fluid, red blood cells, and a large number of inflammatory cells were observed in the alveolar cavity (Fig. 5).

### Balance of Th17 and Treg cells in the spleen

**Fig. 6:**
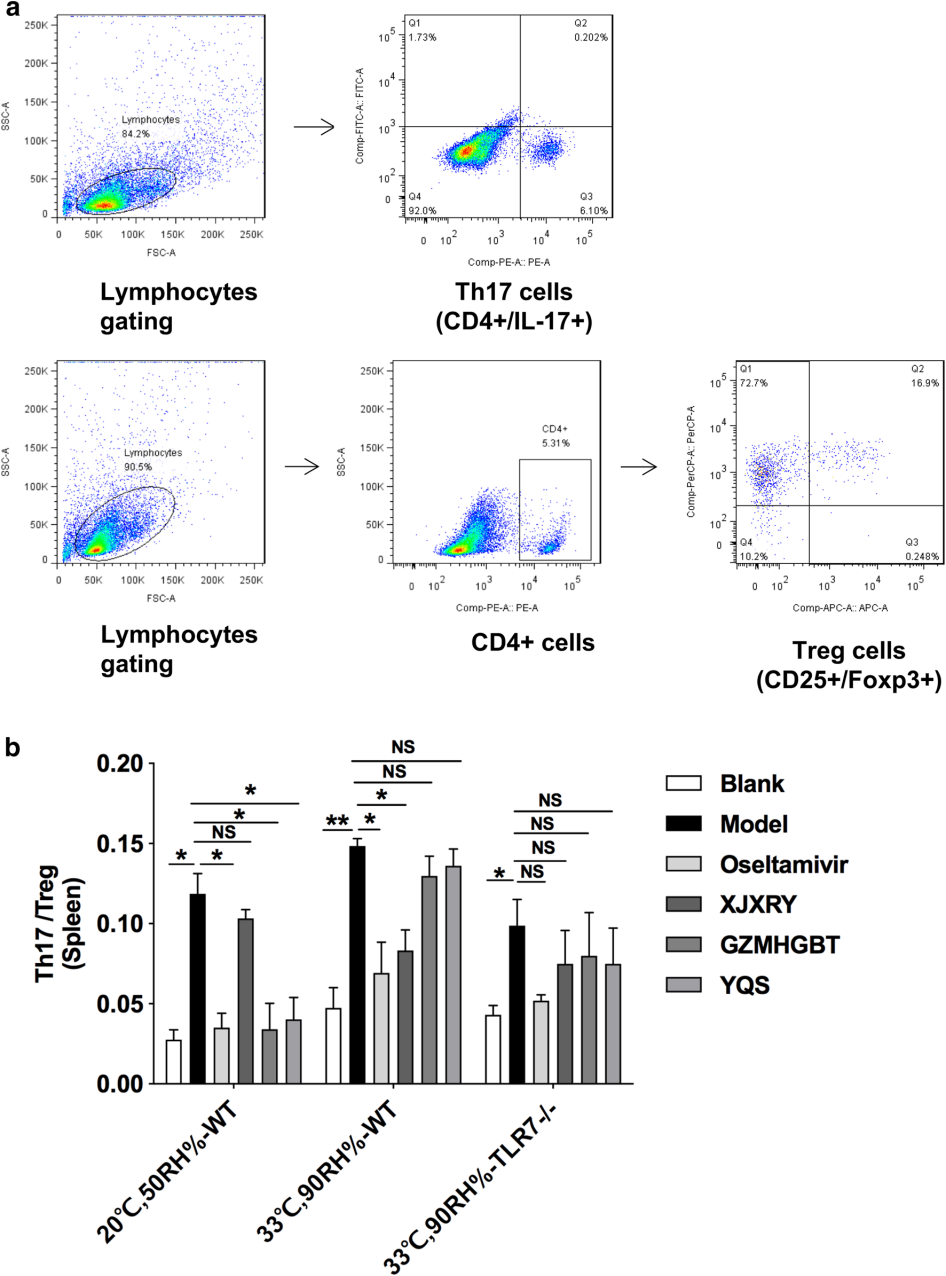
Profiling of Th17 cells and Treg cells in splenocytes of mice. Flow cytometry of CD4+ T cell subsets (**a**). Changes in the proportions of Th17/Treg cell (**b**). This figure shows representative results of experiments with three mice in each group. Statistical analysis was performed by one-way ANOVA. P* = P value for Bonferroni correction. *P < 0.05, **P < 0.01, ***P < 0.001

Under normal conditions (F_(5,12)_ = 11.86, P < 0.0001), compared with the virus group, the GZMHGBT group in WT mice the ratio of Th17 cells and Treg cells in the spleen was significantly decreased (P < 0.05). In the hot and humid (F_(5,12)_ = 10.34, P = 0.0005), XJXRY group was significantly lower than that of the virus group (P < 0.05). In TLR7^−/−^ mice (F_(5,12)_ = 9.23, P < 0.0001), compared with the virus group, only the positive control drug oseltamivir group Th17/Treg cell ratio was reduced (Fig. 6).

### Changes in expression of TLR7, MyD88 and NF-κB p65 mRNA in lung tissue

**Fig. 7:**
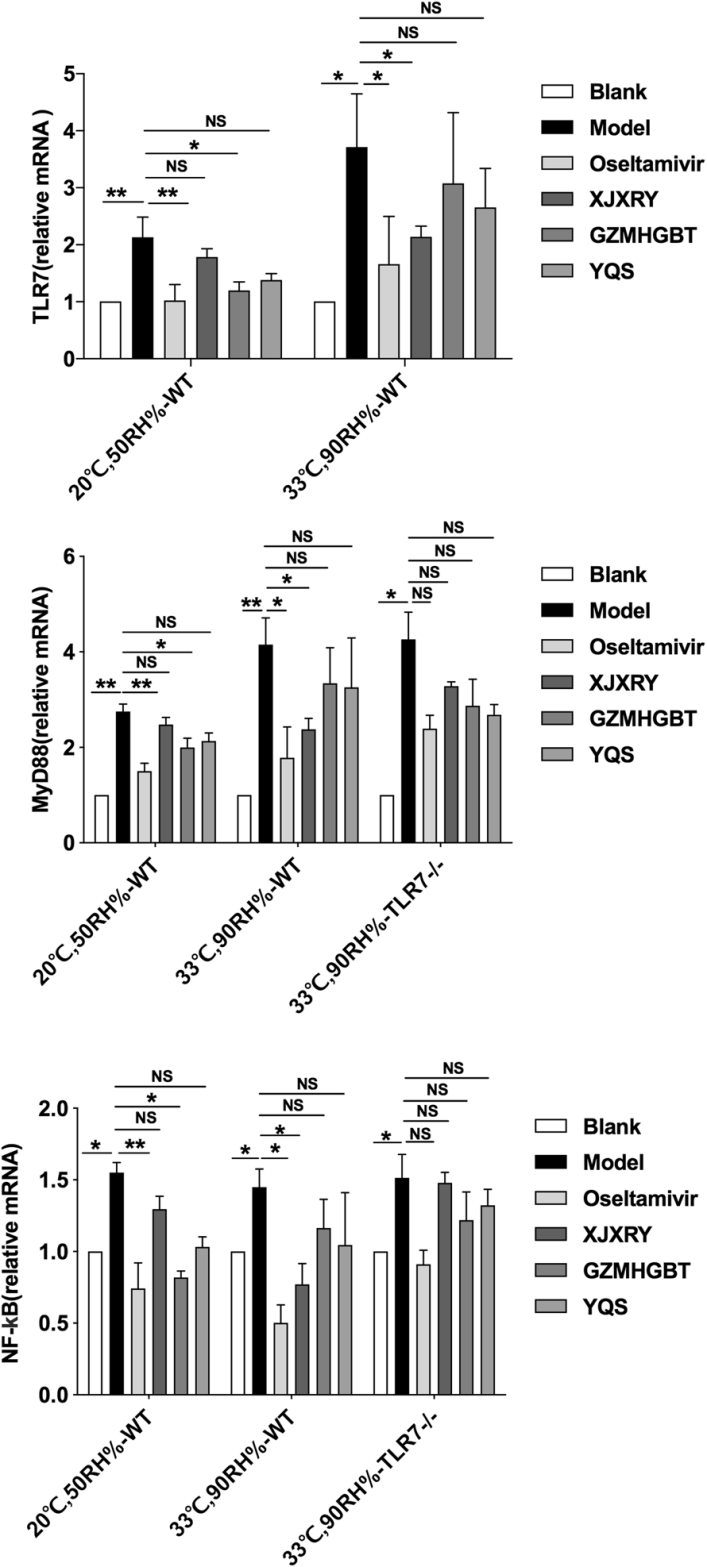
The effect of different drugs on the expression of mRNA in TLR7/NF-κB signaling pathway in lung tissue of influenza virus infected mice. Statistical analysis was performed by one-way ANOVA. P* = P value for Bonferroni correction. *P < 0.05, **P < 0.01, ***P < 0.001

RT-qPCR results showed that the expression of TLR7, MyD88 and NF-κB p65 mRNA in the lung tissue of the virus model group was significantly higher than that of the control group on day 17 (P < 0.05). Whether in normal or hot and humid environments, the mRNA levels of TLR7, MyD88, and NF-κB p65 in the lungs of oseltamivir mice were lower than those in the virus group (P < 0.05). The expression of TLR7 (F_(5,12)_ = 14.06, P = 0.0001), MyD88 (F_(5,12)_ = 10.07, P < 0.0001), and NF-κB p65 (F_(5,12)_ = 10.71, P = 0.0004) mRNA in lung tissue of WT mice in the normal environment, GZMHGBT group was significantly lower than that in the model group (P < 0.05). In WT mice exposed to the hot and humid environment, the expression levels of TLR7 (F_(5,12)_ = 4.78, P = 0.012), MyD88 (F_(5,12)_ = 9.32, P = 0.0009), and NF-κB p65 (F_(5,12)_ = 7.93, P = 0.0014) mRNA in the XJXRY group were significantly different from those in the virus group (P < 0.05), but not in the TLR7 knockout mice. There was no statistical difference between the three treatment groups and the virus group in the TLR7 knockout mice (Fig. 7).

### Changes in the expression of TLR7, MyD88 and NF-κB p65 protein in lung tissue

**Fig. 8:**
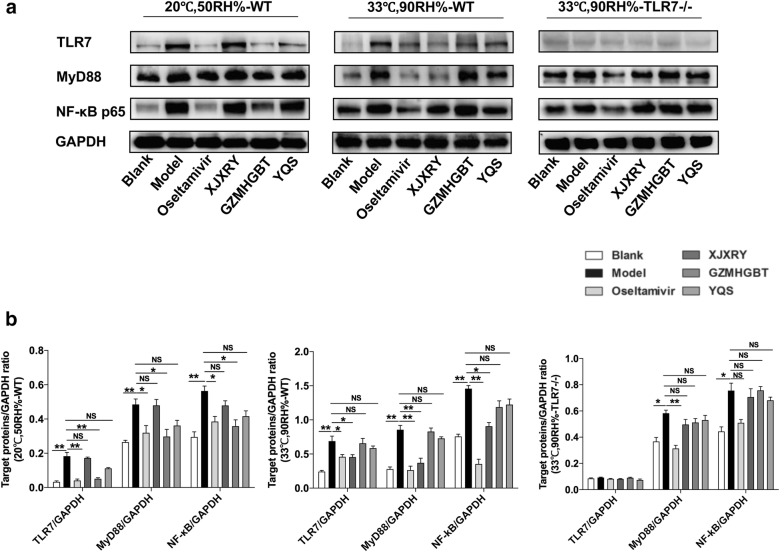
The effect of different drugs on the expression of protein level in TLR7 and NF-κB signaling pathway in lung tissue of influenza virus infected mice. **a** Proteins were evaluated by western blotting assay. **b** Quantification of TLR7, MyD88, NF-κB protein was detected by densitometric analysis. This figure shows representative results of experiments with three mice in each group. Statistical analysis was performed by one-way ANOVA. P* = P value for Bonferroni correction. *P < 0.05, **P < 0.01, ***P < 0.001

Western blot results revealed that the expression levels of TLR7, MyD88 and NF-κB p65 protein in the lung tissue of the virus model group were significantly higher than those of the control group on day 17 (P < 0.05). Under different circumstances, the expression of TLR7, MyD88, and NF-κB p65 protein in the lung tissue of oseltamivir-treated mice was lower than that of the virus group (P < 0.05). The expression levels of TLR7 (F_(5,12)_ = 39.38, P < 0.0001), MyD88 (F_(5,12)_ = 7.83, P = 0.0017), and NF-κB p65 (F_(5,12)_ = 9.49, P = 0.0007) protein in lung tissue of WT mice exposed to the normal environment, GZMHGBT group was significantly lower than those in the model group. In WT mice exposed to hot and humid environments, the expression levels of TLR7 (F_(5,12)_ = 11.43, P = 0.0003), MyD88 (F_(5,12)_ = 28.38, P < 0.0001), and NF-κB p65 (F_(5,12)_ = 36.05, P < 0.0001) protein in XJXRY group were significantly different from those in the virus group (P < 0.05). There was no statistical difference among the three groups treated with Chinese medicine compared with the virus group in the TLR7 knockout mice (Fig. 8).

## Discussion

Chinese medicine has a long history and rich experience in the prevention and treatment of influenza. As early as the 1970s, research investigating single Chinese medicines to treat influenza virus infection has been performed in China. Previously, most scholars primarily conducted drug research and development focused on the direct inhibition of influenza virus using Chinese herbal compounds. In recent years, however, we have gradually changed the perspective of research and have used Chinese medicines to indirectly exert antiviral effects by enhancing immune function. Chinese medicines have a unique effect on the prevention and treatment of influenza, which not only relieves symptoms but also regulates the patient’s own immune function. Traditional Chinese medicine plays a role in targeting the influenza virus by inhibiting virus replication, regulating immune function, and improving blood circulation, antipyretic, analgesia, and its antibacterial and anti-inflammatory properties.

Lingnan is near the South China Sea and has a subtropical marine climate. The temperature is hot and humid, the groundwater resources are abundant and the water level is high in the offshore area, and the surface water area is wide because of the perennial rainy weather [12]. This geographical and climatological feature leads to damp fever. Its unique geographical, environmental and climatic characteristics are more suitable for recognizing damp-heat syndrome than those in Central Plains. At present, the study of damp-heat syndrome model is relatively stable and mature. Many scholars believe that it is more reasonable to simulate the humid and hot environment by simultaneously increasing humidity and temperature [13]. Studies have shown that exogenous dampness pathogen is directly related to the infection of viruses, bacteria and other pathogens. The rats were naturally ill by modeling the moist heat environment. Through the detection of their endocrine, pathological morphology, immunity, intestinal bacteria and other indicators, it is considered that the scientific connotation of dampness and heat is a comprehensive concept which combines seasonal climate environment, biological pathogenic factors and organism reflection [14]. It was found that these patients were commonly found in damp syndrome, thus confirming that there was a certain correlation between respiratory tract diseases and dampness by observing the clinical manifestations of patients with respiratory tract virus infection [15]. At the same time, it further indicates that the immune function of patients after infection is reduced, the accumulation of free radicals in the body and the elimination ability are not balanced. After the application of Chinese herbal medicine for removing dampness, not only the virus is directly inhibited, but also the overall immune function of the body is regulated.

Influenza is a seasonal infectious disease, and belongs to the initial category of exogenous syndromes in traditional Chinese medicine. Traditional Chinese medicine has three basic methods for treating exterior syndromes: cool acrid exterior-resolution; warm acrid exterior-resolution; and clearing damp-relieving superficies However, under different circumstances, the efficacy of three traditional Chinese medicines for treating influenza may be different. These three methods have been widely used clinically for the treatment of upper respiratory tract infections with satisfactory results. However, the efficacy of three traditional Chinese medicines for treating influenza may be different under different circumstances. We used three drugs to represent these three methods: YQS, GZMHGBT, and XJXRY. The effects of these three drugs in the treatment of mice with influenza were compared under normal conditions to observe the incidence of pneumonia in influenza virus-infected mice and the intervention effect on influenza pneumonia in the humid and hot environment. The aim of this study was to investigate the immunological mechanisms of three traditional Chinese medicines in the treatment of mice infected with influenza virus FM1 in normal environment and hot and humid environment, and the effects on TLR7/NF-κB signaling pathway in lungs of mice. We further explain the scientific principle of “treating the same disease with different methods” from the perspective of “environment-organism-pathogen”, and provide theoretical basis and technical platform for research and development of related anti-influenza medicines in the future.

Experimental results revealed that in the humid and hot environment model, the weight of the mice was not significantly affected. In the hot and humid environment, XJXRY clearly exhibited therapeutic effects. Under normal circumstances, GZMHGBT can effectively reduce the lung index of mice with influenza pneumonia and extend the survival time of the animals. XJXRY originated from Wu Jutong’s “Wen Bing Tiao Bian.” The prescription is composed of five herbs including honeysuckle, forsythia, Magnolia, Elsholtzia, and lentil flower. It has the effect of dispelling heat and relieving dampness, and has a significant effect on the symptoms of cold and high humidity in the summer. In pharmacology, the heat-clearing actions of *Forsythiae fructus* are based on the anti-inflammatory and antioxidant properties of lignans and phenylethanoid glycosides. The detoxifying effects are attributed to the antibacterial, antiviral and anti-cancer activities of *Forsythiae fructus*. In addition, the remarkable anti-inflammatory and antioxidant capacities of *Forsythiae fructus* contribute to its anti-cancer and neuroprotective activities [16]. Experiments have shown that phillyrin isolated from Forsythia has potential protective effects against infection caused by the influenza A virus [17]. Honeysuckle is known as an “antibiotic” in Chinese medicine. Modern pharmacological studies have shown that honeysuckle has clear anti-inflammatory, anti-bacterial, anti-viral, anti-oxidation, liver protection, anti-tumor and other properties, in which chlorogenic acid has an anti-influenza virus effect and inhibition of neuraminidase [18], which is one of the main components of honeysuckle. Elsholtzia contains flavonoids and coumarins, and has the functions of heat clearing, wetting, antibacterial, anti-inflammatory and other pharmacological actions [19]. Honokiol in *Magnolia officinalis* also has the effect of inhibiting virus replication [20, 21]. In the present study, XJXRY clearly demonstrated therapeutic effects in mice infected with influenza in a hot and humid environment, and reduced pulmonary inflammation in normal WT mice, but had no significant effect on TLR7^−/−^ mice. Therefore, we speculate that XJXRY may exert anti-influenza and anti-inflammatory effects by down-regulating key factors (i.e., TLR7, MyD88 and NF-κBp65) of the TLR7 signaling pathway.

Recent studies have shown that the balance between Th17 cells and FOXP3+ regulatory T cells (Tregs) is of great significance. Interleukin (IL)-17 secreted by Th17 cells can induce IL-6, IL-8, granulocyte colony-stimulating factor and prostaglandin E2 in epithelial cells, endothelial cells, fibroblasts, and stromal cells. IL-17 also enhances the function of IL-1 and tumor necrosis factor (TNF) on the cell surface and the expression of intercellular adhesion molecule-1 [22]. Therefore, this cytokine plays a role in promoting the inflammatory response. A recent surprising discovery was that the combination of IL-6 and TGF-β can induce the conversion of mouse T cells to produce IL-17 (Th17) [23, 24]. Tregs control the reactivity of self-reactive T cells that are not eliminated in the thymus, and are responsible for maintaining the immune system in homeostatic balance [25]. Cells expressing Foxp3+ transcription factors are essential for maintaining immune homeostasis [26]. Endogenous TGF-β produced by Foxp3+CD4+CD25+ Treg cells enable IL-6 to convert some of these cells to Th17 cells [27]. FOXP3+ Treg is a CD4+ T cell with immunosuppressive function, and is currently the most studied. They have the strongest inhibitory function, cover the most extensive inhibition, can prevent the activation of autoreactive T cells, inhibit the occurrence of autoimmune and allergic diseases and, thus, play an important role in maintaining autoimmune tolerance [28]. An imbalance of FOXP3+ Treg/Th17 cells may cause the onset of autoimmune and allergic diseases [29, 30]. We detected a proportion of Th17/Tregs in the immune cells of spleen tissue in mice using flow cytometry, and found that the value of Th17/Tregs in the virus control group increased compared with the normal control group, indicating that infection with influenza virus (FM1 strain) could promote the direct differentiation of CD4+ T cells to Th17 cells. The present study demonstrated that the ratio of Th17/Tregs in WT mice of YQS and GZMHGBT have a certain degree of down-regulation under normal environment. However, under hot and humid environment, XJXRY could alleviate the inflammatory reaction and reduce the occurrence of autoimmune injury.

The positive control drug in this experiment is oseltamivir, a neuraminidase inhibitor (NAI), which is the mainstream drug for the treatment of influenza. It inhibits NA activity and thus effectively inhibits the replication and spread of various influenza virus strains. For example, the activity of glycoside hydrolase is to cleave the glycosidic linkage of neuraminic acid to reduce viral transmission and reinfection. Existing scholars have found that oseltamivir is effective in early antiviral therapy [31, 32]. However, further clinical trials and basic studies have shown that delayed treatment with NAIs (more than 48 h after onset) is still effective in preventing influenza infection [33, 34]. It can be seen from our experiments that oseltamivir is very effective against influenza virus-infected mice in normal environment and hot and humid environment, and knockout of TLR7 gene did not completely affect its ability to inhibit virus replication. However, in recent years, researchers have found and confirmed that influenza B and H5N1 viruses are resistant to oseltamivir and have more side effects. Compared with it, the traditional Chinese medicine compound not only has low toxicity, multiple targets, but also is resistant to drug resistance and side effects, and can also regulate the body’s immune response as a whole.

## Conclusion

In conclusion, this study confirmed that in normal environments, GZMHGBT demonstrated significant inhibitory effects on influenza virus A (FM1) infection in mice. In hot and humid environments, XJXRY exhibited a good therapeutic effect on mice infected with the influenza A virus (FM1 strain). It can significantly reduce lung inflammation in mice, and exhibit anti-influenza and anti-inflammatory properties by down-regulating the expression of key factors in the TLR7 signaling pathway.

## Data Availability

The data and materials generated or analyzed during this study are available from the corresponding author on reasonable request.

## References

[1] Tam VC, Quehenberger O, Oshansky CM, Suen R, Armando AM (2013). Lipidomic profiling of influenza infection identifies mediators that induce and resolve inflammation. Cell.

[2] Zhang T, Xiao M, Wong CK, Mok KC, Zhao X (2018). A traditional multi-herb formulation, exerts anti-influenza effects in vitro and in vivo via neuraminidase inhibition and immune regulation. BMC Complement Altern Med.

[3] Neumann G, Noda T, Kawaoka Y (2009). Emergence and pandemic potential of swine-origin H1N1 influenza virus. Nature.

[4] Regoes RR, Bonhoeffer S (2006). Emergence of drug-resistant influenza virus: population dynamical considerations. Science.

[5] Janeway CA (1989). Approaching the asymptote? Evolution and revolution in immunology. Cold Spring Harb Symp Quant Biol.

[6] Reizis B, Bunin A, Ghosh HS, Lewis KL, Sisirak V (2011). Plasmacytoid dendritic cells: recent progress and open questions. Annu Rev Immunol.

[7] Akira S, Uematsu S, Takeuchi O (2006). Pathogen recognition and innate immunity. Cell.

[8] Kawai T, Akira S (2010). The role of pattern-recognition receptors in innate immunity: update on Toll-like receptors. Nat Immunol.

[9] Deng ZJ (2003). Formulations.

[10] Zhang BG, Liu QF (2012). Pharmacological research and clinical application of Guizhi-Mahuang Geban-Tang. Chin Trad Pat Med.

[11] Xie JJ, Sun YZ, Gao S, Zhou LL, Li LY, Huang N (2017). HPLC fingerprint for Xinjiaxiangruyin standard decoction. J Int Pharm Res.

[12] Zhou DW, Xu ZW (2017). Formation and academic characteristics of damp-heat disease in Lingnan Medicine. J Basic Chin Med.

[13] Tang XC, Peng SQ (2000). Correlation study between respiratory virus infection and wetness. Chin J Inf TCM.

[14] Zhong L, Chen JH, Wu SJ (1999). The effect of multiple factors on erythrocyte-immunity function in dampness-heat model rats of epidemic febrile disease. Chin J Immunol.

[15] Wang J, Chen YH, Zhao ZL (2002). Produce animal model on wetness–heatness syndrome of seasonal febrile disease. Pharm J Chin People’s Liberation Army.

[16] Wang Z, Xia Q, Liu X, Liu W, Huang W, Mei X, Luo J, Shan M, Lin R, Zou D, Ma Z (2018). Phytochemistry, pharmacology, quality control and future research of *Forsythia suspensa* (Thunb.) Vahl: a review. J Ethnopharmacol.

[17] Qu XY, Li QJ, Zhang HM, Zhang XJ, Shi PH, Zhang XJ, Yang J, Zhou Z, Wang SQ (2016). Protective effects of phillyrin against influenza A virus in vivo. Arch Pharm Res.

[18] Ding Y, Cao Z, Cao L, Ding G, Wang Z, Xiao W (2017). Antiviral activity of chlorogenic acid against influenza A (H1N1/H3N2) virus and its inhibition of neuraminidase. Sci Rep.

[19] Ding CX, Ju JL (2005). Research advance of the chemical component and pharmacological action of Elsholtzia. Shanghai J Trad Chin Med.

[20] Fang CY, Chen SJ, Wu HN, Ping YH (2015). Honokiol, a lignan biphenol derived from the magnolia tree, inhibits dengue virus type 2 infection. Viruses..

[21] Lan KH, Wang YW, Lee WP, Lan KL, Tseng SH, Hung LR, Yen SH, Lin HC, Lee SD (2012). Multiple effects of Honokiol on the life cycle of hepatitis C virus. Liver Int.

[22] Aggarwal S, Gurney AL (2002). IL-17: prototype member of an emerging cytokine family. J Leukoc Biol.

[23] Veldhoen M, Hocking RJ, Atkins CJ, Locksley RM, Stockinger AB (2006). TGFbeta in the context of an inflammatory cytokine milieu supports *de novo* differentiation of IL-17-producing T cells. Immunity.

[24] Bettelli E, Carrier Y, Gao W, Korn T, Strom TB (2006). Reciprocal developmental pathways for the generation of pathogenic effector TH17 and regulatory T cells. Nature.

[25] Horwitz DA, Zheng SG, Gray JD (2008). Natural and TGF-β-induced Foxp3^+^CD4^+^ CD25^+^ regulatory T cells are not mirror images of each other. Trends Immunol.

[26] Kim JM, Rasmussen JP, Rudensky AY (2007). Regulatory T cells prevent catastrophic autoimmunity throughout the lifespan of mice. Nat Immunol.

[27] Xu L, Kitani A, Fuss I, Strober W (2007). Cutting edge: regulatory T cells induce CD4+CD25-Foxp3- T cells or are self-induced to become Th17 cells in the absence of exogenous TGF-beta. J Immunol.

[28] Sakaguchi S, Yamaguchi T, Nomura T, Ono M (2008). Regulatory T cells and immune tolerance. Cell.

[29] Ji L, Zhan Y, Hua F (2012). The ratio of Treg/Th17 cells correlates with the disease activity of primary immune thrombocytopenia. PLoS ONE.

[30] Zhao J, Lloyd CM, Noble A (2013). Th17 responses in chronic allergic airway inflammation abrogate regulatory T cell-mediated tolerance and contribute to airway remodeling. Mucosal Immunol.

[31] Kumar D, Michaels MG, Morris MI (2010). Outcomes from pandemic influenza A H1N1 infection in recipients of solid-organ transplants: a multicentre cohort study. Lancet Infect Dis.

[32] Cao B, Li XW, Mao Y (2009). Clinical features of the initial cases of 2009 pandemic influenza A (H1N1) virus infection in China. N Engl J Med.

[33] Fry AM, Goswami D, Nahar K (2014). Efficacy of oseltamivir treatment started within 5 days of symptom onset to reduce influenza illness duration and virus shedding in an urban setting in Bangladesh: a randomised placebo-controlled trial. Lancet Infect Dis.

[34] Li Z, Li R, Li J (2016). Efficacy of delayed treatment of China-made Peramivir with repeated intravenous injections in a mouse influenza model: from clinical experience to basal experiment. BMC Infect Dis.

